# Combination ART-Induced Oxidative/Nitrosative Stress, Neurogenic Inflammation and Cardiac Dysfunction in HIV-1 Transgenic (Tg) Rats: Protection by Mg

**DOI:** 10.3390/ijms19082409

**Published:** 2018-08-15

**Authors:** I. Tong Mak, Joanna J. Chmielinska, Christopher F. Spurney, William B. Weglicki, Jay H. Kramer

**Affiliations:** 1 Department of Biochemistry and Molecular Medicine, The George Washington University Medical Center, Washington, DC 20037, USA; phyjch@gwu.edu (J.J.C.); wweg@gwu.edu (W.B.W.); phyjhk@gwu.edu (J.H.K.); 2 Division of Cardiology, Children’s National Medical Center, Washington, DC 20010, USA; cspurney@cnmc.org

**Keywords:** HIV-transgenic rat model, combined antiretroviral therapy (cART), oxidative stress, nitrosative stress, neurogenic inflammation, cardio-renal dysfunction, magnesium supplementation

## Abstract

Chronic effects of a combination antiretroviral therapy (cART = tenofovir/emtricitatine + atazanavir/ritonavir) on systemic and cardiac oxidative stress/injury in HIV-1 transgenic (Tg) rats and protection by Mg-supplementation were assessed. cART (low doses) elicited no significant effects in normal rats, but induced time-dependent oxidative/nitrosative stresses: 2.64-fold increased plasma 8-isoprostane, 2.0-fold higher RBC oxidized glutathione (GSSG), 3.2-fold increased plasma 3-nitrotyrosine (NT), and 3-fold elevated basal neutrophil superoxide activity in Tg rats. Increased NT staining occurred within cART-treated HIV-Tg hearts, and significant decreases in cardiac systolic and diastolic contractile function occurred at 12 and 18 weeks. HIV-1 expression alone caused modest levels of oxidative stress and cardiac dysfunction. Significantly, cART caused up to 24% decreases in circulating Mg in HIV-1-Tg rats, associated with elevated renal NT staining, increased creatinine and urea levels, and elevated plasma substance P levels. Strikingly, Mg-supplementation (6-fold) suppressed all oxidative/nitrosative stress indices in the blood, heart and kidney and substantially attenuated contractile dysfunction (>75%) of cART-treated Tg rats. In conclusion, cART caused significant renal and cardiac oxidative/nitrosative stress/injury in Tg-rats, leading to renal Mg wasting and hypomagnesemia, triggering substance P-dependent neurogenic inflammation and cardiac dysfunction. These events were effectively attenuated by Mg-supplementation likely due to its substance P-suppressing and Mg’s intrinsic anti-peroxidative/anti-calcium properties.

## 1. Introduction

Since the introduction of highly active antiretroviral therapy (HAART, or combination antiretroviral therapy, cART), HIV-infection has become a more manageable chronic disease [1,2,3,4,5]. However, as the life expectancy of HIV-patients lengthens, the frequency of cardiovascular disease (CVD) has become a major concern, partly due to the chronic HIV infection itself, and partly due to the use of HAART [1,2,3]. HAART, which is comprised of different classes of agents, may contribute varying degrees of toxicity and metabolic side-effects that can enhance the risk of CVD. Among the major groups of HAART are nucleoside reverse transcriptase inhibitors (NRTI), Non-Nucleoside reverse transcriptase inhibitors (NNRTI) and protease inhibitors (PI) [4]. We previously showed that mono-treatment of normal rats with the NRTI, zidovudine (AZT), the NNRTI, efavirenz (EFV), or the PI, ritonavir (RTV), each caused varying degrees of oxidative stress [6,7,8]; of these 3 different classes of agent, AZT displayed the most oxidative activity in the rat model [7]. However, RTV, which in addition to causing endothelial oxidative dysfunction [9,10], also induces prominent lipogenic effects [8]. In addition to HAART-related cardiovascular (CV) effects, it is also recognized that HIV infection itself, produces CVD and renal side effects which may partly be due to persistent immune/inflammatory activities [10,11,12]. Current HAART therapy employs combination regimens consisting of different anti-retroviral agents in order to lessen the chance of drug resistance [4,13]. The most common first line HAART regimens include 2-NRTIs plus a boosted PI or an integrase inhibitor [4]. In this study, we focus on the cardiovascular toxicity of a cART consisting of Truvada (a 2-NRTI combination of tenofovir disoproxil fumarate (TDF)+emtricitabine) plus RTV-boosted atazanavir (PI: ATV), which until 2016 was considered a first line HAART regimen. To determine the effects of HIV expression, we chose to use the well-established HIV-1 Transgenic (Tg) rat model, which expresses 7 of the 9 HIV genes, except for gag and pol which are responsible for infectivity [11,14,15]. The remaining 7 genes carry most of the proteins responsible for the eventual development of AIDS symptoms, but without the complications of infectivity [14,15,16].

While examining micronutrient status in HIV-1 infected patients during the pre-HAART era, Bogden et al. [17] discovered that 30% of these individuals had significant hypomagnesemia; concurrently, Brod-Miller [18] reported that 30–60% of the HIV-patients displayed hypomagnesemia. Later clinical studies [19,20,21,22] further showed that HIV-1 infected patients treated with HAART displayed significantly lower circulating Mg in at least 1/3 of the patients, confirming the notions that both HIV infection and HAART treatment contributed in some way to excessive loss of Mg. However, the contribution to Mg loss from HIV-1 infection and from specific ART treatments was unclear. The possibility exists that HIV-1 infection combined with certain ART regimen leads to a significant impact on renal Mg-wasting. This current study investigates the hypothesis that certain HAART agents such as Truvada may cause cardiovascular toxicity due to a synergistic renal injury during HIV infection, and that both conditions together may enhance Mg-wasting/hypomagnesemia, leading to neurogenic inflammation, systemic oxidative/nitrosative stress, cardiac inflammation and contractile dysfunction. It is also hypothesized that this combined HAART and HIV related-toxicity is correctible with Mg-supplementation.

## 2. Results

### 2.1. Effects of HIV-1 Expression ± cART on Body Weight Gain, Food and Drug Consumption

As presented in Table 1, based on per kg body weight, the average food intake for the four groups receiving the normal Mg diet (A1–A4) at 18 weeks was comparable on a daily basis as well as throughout the entire study period. The food intakes for the Mg-supplemented groups (B1–B4) at 18 weeks were also comparable to each other and to their normal Mg counterparts. Based on measured food consumption, the average individual cART component dosages for normal Mg groups were estimated to be: TDF or ATV: 15.8 ± 0.6 mg, emtricitabine: 10.4 ± 0.5; and RTV: 5.2 ± 0.3 mg/kg body wt/day. For the Mg-supplemented groups, the parallel dosages were estimated to be: TDF or ATV: 15.7 ± 1.3 mg; emtricitabine: 10.3 ± 0.8; and RTV: 5.2 ± 0.4 mg/kg/day. Overall, the drug dosages for the control and Tg rats were comparable, and no significant differences were detected between normal Mg and their Mg supplemented counterpart. However, as a first sign of stress due to cART administration, the rate of weight gain by Tg rats (at 18 weeks) was 23% lower than controls, which is statistically significant (*p* > 0.01). Interestingly, cART treatment did not lower the rate of weight gain in normal Mg control rats. Moreover, it was remarkable that cART had no adverse effect on weight gain of the HIV-Tg rats concurrently receiving the Mg supplemented diet.

### 2.2. Effects of HIV-1 Expression ± cART on Indices of Oxidative Stress

**Figure 1:**
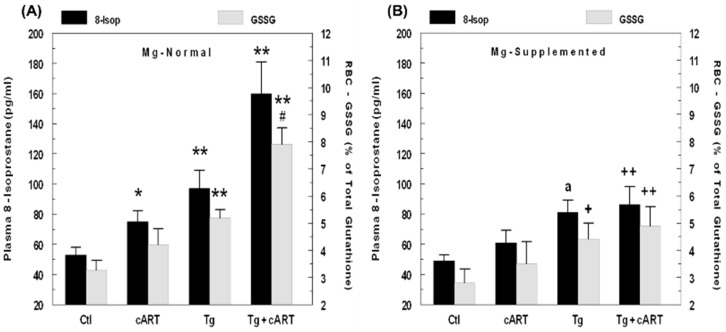
HIV-1 expression and cART–mediated elevations in: Plasma 8-isoprostane and RBC GSSG levels in 6-week normal Mg rats (**A**); and attenuation by Mg supplemented (6-fold higher) diet (**B**). Values are means ± SE = 5 rats/group. * *p* < 0.05 and ** *p* < 0.01 vs. respective Ctl. ^#^ *p* < 0.05 vs. Tg alone; ^a^ *p* = 0.055 vs. Mg-normal Tg alone; ^+^ *p* < 0.05 and ^++^ *p* < 0.01 vs. Mg-normal counterparts in panel A.

As an index of systemic oxidative stress caused by cART, plasma levels of F2-like isoprostanes (as 8-isoprostane), which are derived from nonenzymatic peroxidation of polyunsaturated fatty acids [23], were determined. This index is often used to assess the impact of HAART or HIV-1 virus in patients [23,24]. Figure 1A shows the plasma levels of isoprostane in rats after 6 weeks of treatment: cART treatment alone led to a modest 41% increase in isoprostane in normal Mg control rats; HIV-Tg alone exhibited a 92% higher isoprostane level; and cART treatment of Tg rats further elevated isoprostane levels 2.8-fold compared to normal Mg controls. In data not shown, isoprostane levels from 12- and 18-week-Tg-treated rats receiving cART were 2.5-fold and 2.3-fold higher than controls, respectively, whereas Tg alone maintained about 90% above control levels after 12 and 18 weeks. As a secondary indicator of systemic oxidative stress, we determined changes in the RBC GSSG/GSH ratio (Figure 1A). Under normal condition, the RBC GSSG content for control rats was low (about 3 %); cART increased this slightly (N.S) to 3.8% in controls. The GSSG level from the HIV-Tg alone was significantly higher (5.3%, *p* < 0.01) versus the control, and with cART, this was elevated further to 8% (*p* < 0.01). These findings indicate that enhanced oxidative stress was occurring in Tg rats receiving cART. Dietary Mg supplementation not only reduced the elevations in isoprostane levels in the cART-treated control and Tg only groups, but the much higher increases observed in cART-treated Tg rats were also substantially reduced (Figure 1B). Concomitantly, the corresponding rise in the RBC GSSG content in cART-treated Tg rats was normalized as well.

### 2.3. Effect of cART on Neutrophil Activation

**Figure 2:**
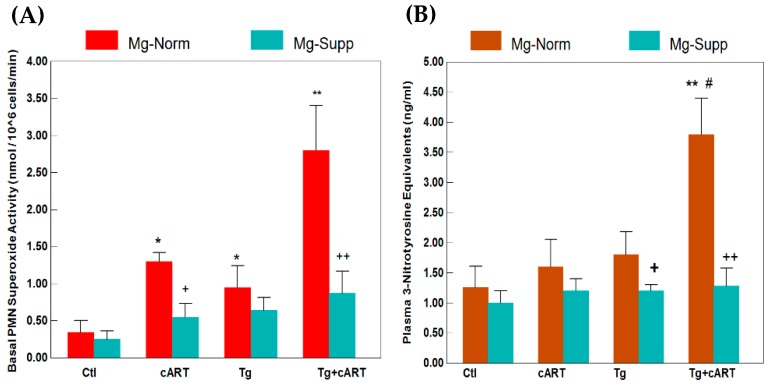
cART-enhanced basal superoxide production in circulating neutrophils (**A**), and elevated plasma 3-nitrotyrosine (**B**) in HIV-1 Tg rats receiving 18 weeks of cART treatment: Effect of Hi Mg diet. Values are means ± SE = 5 rats/group. * *p* < 0.05 and ** *p* < 0.01 vs. respective Ctl; ^#^ *p* < 0.05 vs. Mg-normal Tg alone. ^+^ *p* < 0.05 and ^++^ *p* < 0.01 vs. Mg-normal counterparts.

To seek corroborative evidence of increased ROS stress, we isolated the neutrophils from the whole blood and determined the superoxide generating activity of these endogenous inflammatory cells. Figure 2A demonstrates basal superoxide generating activities from 18-week treated rats of different groups. Compared to the untreated controls, basal activity was moderately elevated by 2.8-fold in PMNs from HIV-Tg, and was 3.8-fold higher in the cART–treated controls; however, substantially higher activity was observed in the cART–treated Tg group, which was 8.24-fold higher than untreated controls. Remarkably, concurrent Mg-supplementation suppressed the basal superoxide generating activities in all groups, but the most significant decrease was seen in the cART–treated Tg group receiving the Mg supplement (Figure 2A). However, in data not shown, in the presence of PMA, all groups appeared to be stimulated to comparable extents of activity.

### 2.4. Impact of cART on Plasma and Cardiac Nitrotyrosine (NT) Elevations in Tg and Control Rats on Normal or Mg Supplemented Diets

In addition to oxidative stress, we determined if nitrosative stress, resulting from O_2_^−^ reacting with NO leading to peroxynitrite formation, might also occur in Tg rats. An immunochemical assay for plasma 3-nitrotyrosine levels formed due to peroxynitrite mediated nitration of protein tyrosine moiety was used. As shown in Figure 2B, plasma nitrotyrosine levels at 18 weeks increased by only 33% (NS) and 50% (NS) in the cART-treated control and untreated Tg groups, respectively. However, a dramatic and significant increase (317%, *p* < 0.01) was observed in 18-week in Tg rats receiving cART treatment. Again, concurrent Mg-supplementation lowered plasma nitrotyrosine to control levels under all conditions (Figure 2B).

**Figure 3:**
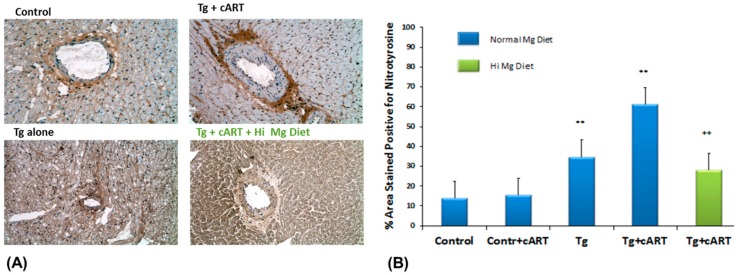
Representative micrographs for 3-nitrotyrosine formation in cardiac ventricular tissue of Ctl & HIV-1 Tg rats receiving cART with or without Mg-supplementation for 18 weeks (**A**); and % area stained positive for 3-nitrotyrosine (**B**). Values are means ± SE = 5 rats/group; ** *p* < 0.01 vs. Ctl, ^++^ *p* < 0.01 vs. Mg-normal Tg + cART.

We examined if nitrosative stress was prevalent at the organ (cardiac) level by assessing the extent of nitrotyrosine formation using rabbit anti-3-nitrotyrosine antibody, which in reaction with secondary antibody, conjugates with horseradish peroxidase and reveals brown staining. As seen in Figure 3A, intense staining for nitrotyrosine (peroxynitrite tissue marker) was observed in peri-vascular connective tissue of ventricles from 18-week cART-treated Tg rats, but staining appeared more diffuse in the other groups. Using ImageJ software, the relative intensity of staining for the different groups was quantified: while heart tissue from cART-treated Tg rats displayed the highest intensity, tissue from untreated Tg also revealed elevated but lower staining intensity for nitrotyrosine (Figure 3B). We found that Mg-supplementation also significantly (*p* < 0.01) reduced tissue nitrotyrosine staining in the 18-week cART-treated Tg group; the staining in the Tg+ Hi Mg group was also attenuated compared to the Tg normal Mg group (see Figure S1, Tables S1 and S2).

### 2.5. Effects of HIV-1 Expression ± cART on Cardiac Function/Hemodynamics

**Figure 4:**
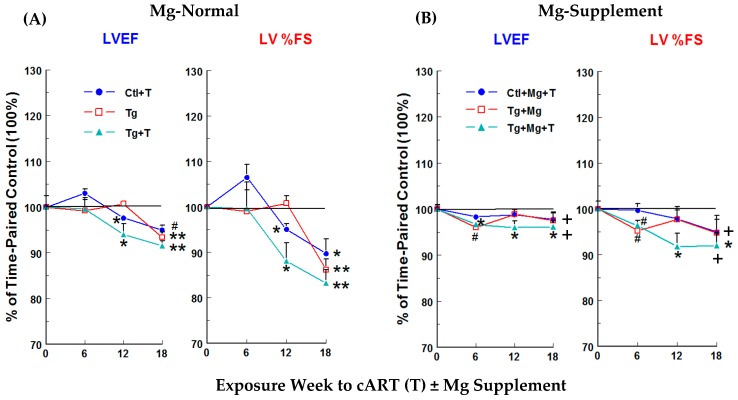
Temporal effects of HIV-1 expression ± cART (T) on LV systolic function of Mg-normal (**A**) and Mg-supplemented (**B**) rats. LV ejection fraction (LVEF) and % fractional shortening (%FS) were monitored by echocardiography for 18 weeks. Values are means ± SE = 5 rats/group. * *p* < 0.05, ^#^ *p* < 0.02, ** *p* < 0.01 vs. Ctl (100%); ^+^ *p* <0.05 vs. Mg-normal counterparts.

**Figure 5:**
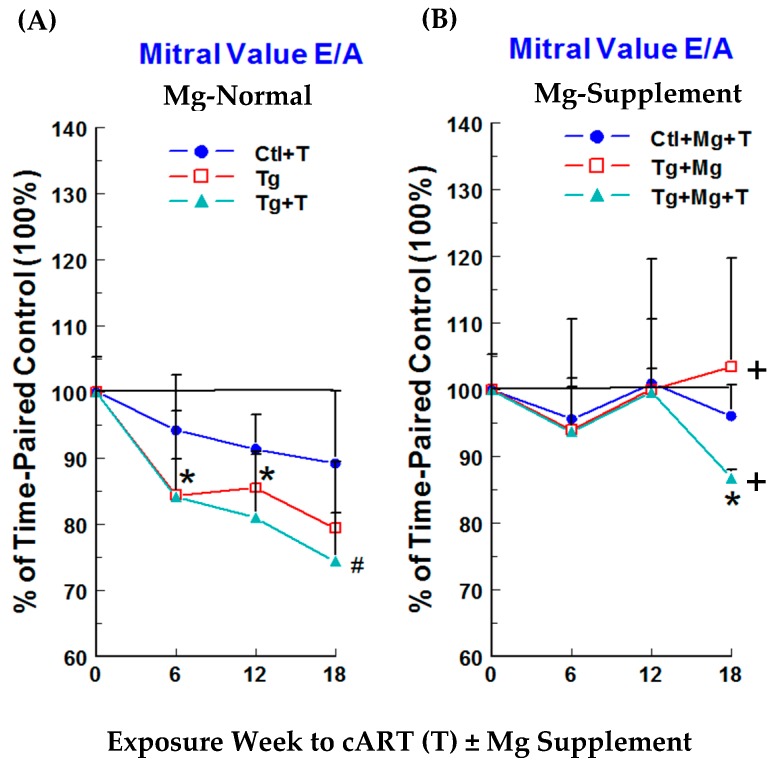
Temporal effects of HIV-1 expression ± cART (T) on LV diastolic function (mitral valve E/A ratio) of Mg-normal (**A**) and Mg-supplemented (**B**) rats during 18 weeks. E/A was monitored by echocardiography. Values are means ± SE = 5 rats/group. * *p* < 0.05, ^#^ *p* < 0.02 vs. Ctl (100%); ^+^ *p* < 0.05 vs. Mg-normal counterparts.

**Figure 6:**
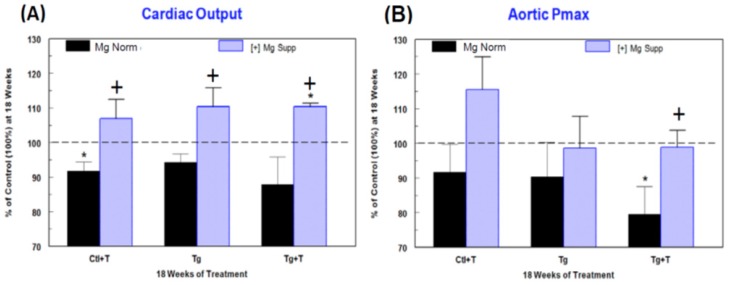
Effects of HIV-1 expression ± cART (T) on (**A**) cardiac output and (**B**) aortic pressure maximum of Mg-normal and Mg-supplemented rats after 18 weeks. These parameters were monitored by echocardiography. Values are means ± SE = 5 rats/group. * *p* < 0.05 vs. Ctl (100%); ^+^ *p* < 0.05 vs. Mg-normal counterparts.

Cardiac functional changes were monitored by non-invasive echocardiography. No significant changes in left ventricular (LV) systolic function, monitored as LV ejection fraction (LVEF) and % fractional shortening (% FS), were observed at 6 weeks for Ctl + cART treatment (T), Tg alone, or Tg + T rats on Mg-normal diet versus time-matched controls (Figure 4A). Small but significant decreases were observed at 12 weeks for LVEF in Ctl + cART (−2.4%) and Tg + cART (−6%) rats and for % FS in Ctl + cART (−4.9%) and Tg + cART (−11.9%), but no changes were detected in the Tg only group. However, by 18 weeks, each group exhibited significant (*p* < 0.05) systolic dysfunction: LVEF for Ctl + cART (T), Tg, and Tg + cART were −5, −6.6, and −8.5%, respectively; and % FS for Ctl + cART, Tg alone, and Tg + cART were −10.3, −13.8, and −16.8 %, respectively. Decreases in the mitral valve E/A ratio (Figure 5A) were observed at 6, 12 and 18 weeks for Ctl + cART (−5.8 to −10.8%), Tg alone (−14.5 to −20.6%), and Tg + cART (−15.8 to −25.6%), suggesting early signs of LV diastolic dysfunction. Moreover, hemodynamic properties were modestly depressed at 18 weeks for Ctl + cART, Tg alone and Tg + cART groups versus the control. Cardiac output (Figure 6A: CO) was 8.3% (*p* < 0.05), 5.7% (ns), and 12.1% (ns) lower for Ctl + cART, Tg alone and Tg + cART; whereas aortic pressure maximum (Figure 6B: Aortic Pmax) was reduced by 8.3% (ns), 9.7% (ns), and 20.5% (*p* < 0.05) for the Ctl + cART, Tg only and Tg + cART groups.

### 2.6. Mg-Supplementation on Cardiac Function/Hemodynamics of cART−Treated HIV-Tg and Control Rats

The functional integrity of cART-treated Ctl and Tg rats benefited from dietary Mg-supplementation. Protection against loss of systolic and diastolic function was observed in all Mg-supplemented groups. Figure 4B (LVEF & % FS) and Figure 5B (E/A) shows the positive effects of Mg throughout 18 weeks of exposure. At 18 weeks, Mg attenuated the loss of LVEF by 48–62%, LV % FS by 51–62%, and E/A by 48–100% versus their respective Mg-normal values (Figure 4A and Figure 5A). In addition, Mg supplementation at 18 weeks improved cardiac output by 6.9%, 10.3% and 10.2% above Mg-normal control levels (100%) (Figure 6A), and aortic pressure maximum rose by 7.6% or was almost normalized to control values (Figure 6B) for Ctl + cART, Tg only and Tg + cART groups, respectively.

### 2.7. Cardiac Anatomical Parameters and Effects of Mg

In data not shown, two-dimensional M-mode and pulsed Doppler images were used to assess measurements of the left ventricular (LV) posterior wall thickness and internal chamber diameter of 18-week normal Mg rats (Suppl. data). Modest decreases (10–15%) were revealed mainly in the Tg + cART rat hearts for LV posterior wall thickness in the systole and in the thickness of the interventricular septum in diastole (IVSd) and systole (IVSs), suggesting that prolonged cART may lead to the onset of a dilated cardiomyopathy. Decreases of a lesser extent were also observed in the Tg only and control + cART groups. Co-treatment with Mg supplementation prevented much of the declines in these cardiac anatomical parameters caused by HIV-transgenicity ± cART. Thus, dietary Mg supplementation was substantially beneficial against the loss of cardiac systolic and diastolic function, hemodynamic properties and anatomical abnormalities caused by HIV-1 expression plus cART in this experimental model.

### 2.8. Time-Course Changes in Plasma Mg during cART Treatment of Tg and Control Rats

**Figure 7:**
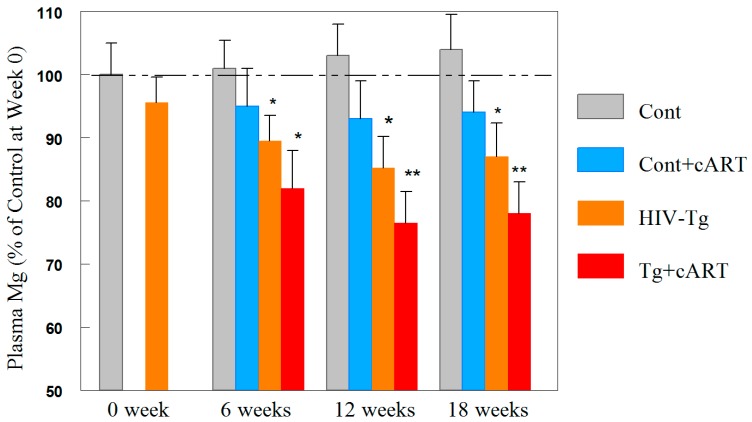
cART promoted hypomagnesemia in HIV-1 Tg rats (up to 18 weeks). Plasma Mg levels were assessed by atomic absorption spectroscopy. The control values at week 0 were measured to be 19.6 ± 1.2 mg/dL, and % of control values are means ± SE = 5 rats/group; * *p* < 0.05, ** *p* < 0.01 vs. Ctl.

Without exception, Mg-supplementation was found to attenuate all of the oxidative/nitrosative stress indices and cardiac dysfunction resulting from cART treatment in both rat strains. Thus, we investigated if cART leads to hypomagnesemia. As presented in Figure 7, prior to the introduction of cART treatment (zero time), there was no significant difference in plasma Mg levels between the control and Tg animals (100 ± 8% versus 97 ± 6%, *n* = 10). However, after 6 weeks of cART treatment, only a slight decrease (−5%, N.S.) was observed in cART-treated controls; a modest decrease was noted in the Tg only group (−10.5%, *p* < 0.05), but a larger decrease (−19%, *p* < 0.05) in blood Mg was detected in cART-treated Tg rats. At 12 weeks, the Tg + cART group displayed the highest decline in circulating Mg (−24%, *p* < 0.01); and at 18 weeks, the decrease remained −21% (*p* < 0.05). In comparison, Tg only rats experienced −14% (*p* < 0.05) and −12% decreases at week 12 and 18, respectively, whereas cART treatment of controls caused only non-significant decreases no greater than 6%.

### 2.9. Kidney Nitrosative Stress and Decreases in Function

**Figure 8:**
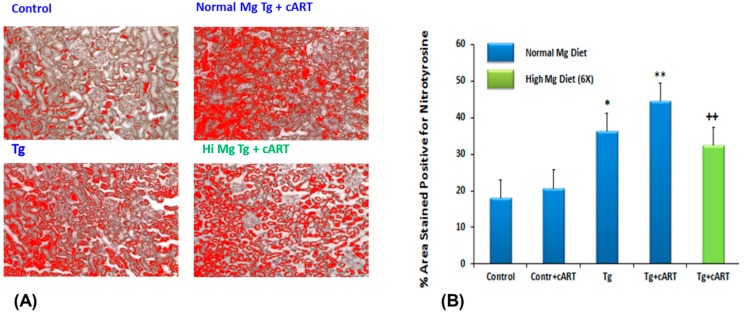
Micrographs showing immunohistochemical staining for 3-nitrotyrosine (NT) in kidneys of Ctl and HIV-1 Tg ± cART ± Mg supplemented (Hi Mg) rats (18 weeks) (**A**). NT staining is selected with red color in the ImageJ program used for quantification and is pre-dominant in kidney cortex; % area stained positive for NT of Tg ± cART ± Mg supplemented rats (**B**). Values are means ± SE = 5 rats/group. * *p* < 0.5; ** *p* < 0.001 vs. Ctl; ^++^ *p* < 0.02 vs. Mg-normal Tg + cART.

To determine if kidney dysfunction might be related to oxidative/nitrosative stress similar to that observed in the heart, we assessed changes in levels of 3-nitrotyrosine in kidneys from each rat group. As shown in Figure 8A, while immunohistochemical staining intensity of 3-nitrotyrosine was only mild in cART-treated control tissue, a significant level of intensity was observed in Tg alone; moreover, even greater staining intensity was revealed in samples from Tg rats receiving cART (Figure 8B). Again, Mg-supplementation effectively suppressed 3-nitrotyrosine staining in the Tg + cART kidneys, and also in the Tg alone group (see Figure S1, Tables S1 and S2).

**Figure 9:**
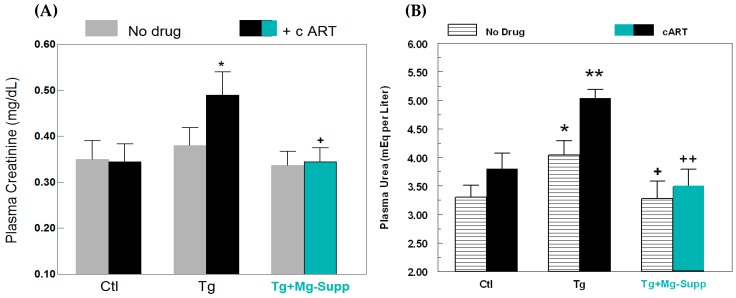
Effects of cART ± Mg—supplementation on (**A**) plasma creatinine levels, and (**B**) plasma urea levels in 18 week Ctl and HIV-1 Tg rats. Values are means ± SE = 5 rats/group. * *p* < 0.05, ** *p* < 0.01 vs. Ctl; ^+^ *p* < 0.05, ^++^ *p* < 0.01 vs. untreated Tg rats with normal Mg.

We also assessed if the observed hypomagnesemia might potentially relate to renal dysfunction, by assessing clearance of plasma creatinine and urea. Figure 9A shows that cART alone had virtually no effect on plasma creatinine levels in control rats after 18 weeks; untreated Tg rats displayed a minor elevation (+9%, NS), but cART treatment of Tg rats led to a significant enhancement (+40%, *p* < 0.05) of plasma creatinine level. As further confirmation of kidney dysfunction, the clearance of plasma urea was evaluated, since it is frequently used as clinical index of renal function. Plasma urea is a principle nitrogenous end-product of protein and amino acid catabolism, which is filtered out of blood by glomeruli. As indicated in Figure 9B, cART treatment only had a mild elevating effect (+15%, NS) on the plasma urea level in control rats, but Tg alone displayed a modest rise of plasma urea (+22.4%, *p* < 0.05). However, cART treatment of Tg rats led to a substantial increase (+53%, *p* < 0.01), which was significantly higher than Tg alone or the control. In keeping with its effects on renal nitrosative stress, concurrent Mg-supplementation substantially attenuated the levels of plasma creatinine (Figure 9A) and urea (Figure 9B) in Tg + cART rats.

### 2.10. Effects of HIV-1 ± cART on Neurogenic Inflammation and Impact of Mg Supplementation

**Figure 10:**
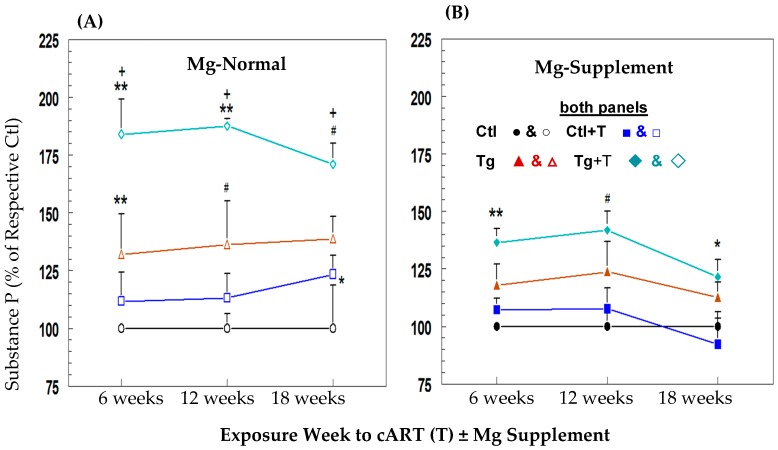
Effects of cART (T) on plasma substance P (SP) level time-courses in control (Ctl) and HIV-1 Tg rats on (**A**) Mg-normal or (**B**) Mg-supplemented diets. SP was assessed by colorimetric ELISA. Values are means ± SE = 5 rats/group for Mg-normal (open symbol) and Mg-supplemented (closed symbol). * *p* < 0.05, ^#^ *p* < 0.02, ** *p* < 0.01 vs. respective Ctl (100%); ^+^ *p* < 0.05 vs. Mg normal time-matched Tg counterparts.

We [25] previously showed that the severity of diet-induced hypomagnesemia was directly proportional to the extent of circulating substance P (SP) elevation and associated ROS stress in a rodent model. Mild to moderate hypomagnesemia was observed in Tg and cART-treated Tg rats after 12 weeks and was sustained throughout 18 weeks. In association, modest elevations (non-significant) in circulating SP levels persisted throughout 18 weeks for the Tg group (+32, +36.3 & +38.8% higher at 6, 12, & 18 weeks versus control), but cART further enhanced circulating SP levels of Tg rats (+84, +87.6, & +71% higher at 6, 12, & 18 weeks) significantly compared to control and their untreated Tg counterpart (Figure 10A). Tg rats placed on the Mg supplemented diet (Figure 10B) exhibited modest declines in circulating SP levels (−44, −34.4 & −67.5% lower at 6, 12 & 18 weeks) compared to the normal Mg Tg group; however, the largest decreases by Mg supplementation were in cART-treated Tg rats (−56, −52, & −69.7% lower SP at 6, 12, & 18 weeks) versus the corresponding Mg-normal Tg rats.

## 3. Discussion

In previous studies, we reported that individual HAART agents such as AZT, EFV, and RTV can cause substantial systemic oxidative stress, cardiac pathology and contractile dysfunction in normal rats [6,7,8]. However, due to HIV mutations and rapid development of drug resistance, use of monotherapy has been largely replaced by combinational ART treatment which is much more effective in suppressing HIV replication and minimizing drug resistance [4,13]. In this study, we employed a clinically relevant combination ART regimen which consisted of 2 NRTIs (Truvada) and 2 PIs (ATV/r). In addition, the dosage used for this ART combination in rats was lower than that prescribed in human equivalence. Due to the higher metabolic rates of rodents, it has been recommended by the US Food and Drug Administration that the equivalent drug dosage for rats and mice should be 6.2- and 11-fold higher [26], respectively, to approach the plasma level equivalence in humans. In the current study, the cART dosage in rats was only 3-fold higher than the human clinical dosage, and thus, was approximately ½ of the prescribed human equivalent dosage. This might explain our observations that cART administered to controls caused only modest levels of oxidative stress, as evidenced by the small elevations in plasma isoprostane and RBC GSSG levels, and the modest neutrophil superoxide production. Using 3-nitrotyrosine formation as a marker for nitrosative stress, the cART regimen caused no change in plasma NT levels, and only small effects on cardiac and renal NT staining in control rats. In association, minimal effects on cardiac function were observed throughout the 18-week experimental period. cART-treated controls also exhibited minimal injury to the kidney, as evidenced by the relatively low plasma creatinine and urea levels. However, when the same low dosage of cART regimen was administered to the HIV-Tg rat, substantially higher increases in systemic oxidative/nitrosative stress were observed, as indicated by the significantly elevated plasma 8-isoprostane, RBC GSSG and plasma NT levels. The prominent increases in NT-staining in the heart and kidney from Tg + cART rats suggested that nitrosative stress was occurring not only systemically, but also in the affected organs. In association, we observed time-dependent significant decreases in both left ventricular systolic (LVEF, %FS) and diastolic (mitral valve E/A ratio) function in the Tg + cART group, with the largest decreases occurring at 18 weeks. HIV-gene expression alone (Tg) did cause some level of oxidative stress as indicated by the moderate elevations in 8-isoprostane and GSSG levels, and the associated intermediate loss of contractile function. The HIV-Tg group also displayed some degrees of nitrosative stress at the systemic and cardiac tissue level. Overall, our findings are consistent with the published observation that HIV gene expression alone can exert systemic oxidative stress that is likely due to the known pro-oxidative properties of gp 120, TAT, and Nef, which are 3 of the remaining 7 genes expressed in HIV-Tg rats [14,15,16].

Previously, we reported that dietary Mg-supplementation protected against cardiovascular toxicity in normal control rats receiving chronic monotherapy with either AZT, ritonavir (RTV) or efavirenz (EFV) [6,7,8]. In the current study, we found that Mg-supplementation effectively suppressed the systemic ROS/RNS stress in the cART treated HIV-1 Tg rats; in association, substantial attenuation of the induced renal and cardiac dysfunctions was observed. It is generally accepted that Mg can function as a “natural calcium blocker” [27,28]. We previously reported that dietary Mg supplementation can substantially increase (33%) total circulating Mg levels in normal rats [7,8,9]; Mg’s anti-calcium effect might depress Ca^2+^-mediated priming and activation of NADPH oxidase in circulating PMNs [29] from cART^−^ treated rats. Moreover, calcium is essential for COX-2 activation as well as PGE_2_ production [30], and suppression of these events by Mg supplementation may reflect its Ca-antagonistic effect. In additional AA experiments, we found that Mg-supplementation did not result in significant changes in plasma calcium levels for the control rats (102 ± 5% vs. 100% ± 3%, N.S.). Likewise, the calcium levels for the Tg rats were 98 ± 3% (N.S.) with normal Mg and 101.5 ± 2.5% (N.S.) with Mg-supplementation compared to controls.

Evidence from our laboratory [31,32,33] and those of others [34,35] suggests that Mg may also exhibit anti-ROS/RNS properties. The protective effects of high Mg^2+^ may partially relate to its competitive displacement of low molecular weight iron (pro-oxidant) from membrane phospholipid binding sites, thereby averting site-specific hydroxyl radical formation [31,32,33]. We suggest that both anti-ROS/RNS and anti-calcium properties of Mg may contribute to the protective effects provided by Mg-supplementation against the synergistic oxidative stress and cardiac dysfunction displayed in cART-treated Tg rats.

We further investigated the underlying mechanism(s) related to the toxicity caused by cART treatment in the Tg rats. During treatment, moderate but significant levels of hypomagnesemia were evident in the cART-treated Tg rats as early as 6 weeks and this proceeded to a maximum extent (−24%) at 12 weeks. After 18 weeks, the plasma Mg level remained persistently low (−21%) compared to the control. It is noteworthy that there were no significant differences in plasma Mg levels between the 3-month-old control and Tg rats prior to cART treatment (week 0). One likely cause of hypomagnesemia might be due to excessive Mg wasting resulting from kidney dysfunction. Tenofovir disoproxil fumarate (TDF) is the main component of Truvada, and is known to concentrate within the kidney tubular mitochondria, where it can exert oxidative stress [36,37]. In a rat study [38], it was concluded that the depletion of cellular antioxidant components such as the glutathione-dependent system, contributed to TDF-induced proximal tubular mitochondria damage as well as increased nitrosative stress. Indeed, our results confirm the occurrence of nitrosative stress in kidneys of cART-treated Tg rats. The potential toxicity of TDF may involve renal tubular dysfunction that ranges from low grade plasma creatinine elevations to significant tubular dysfunction and Fanconi’s syndrome [36,39]. We postulated that TDF-induced renal dysfunction might lead to Mg wasting and hypomagnesemia. The clinical linkage between TDF treatment in HIV patients and Mg-wasting remains unclear due to the fact that both urinary Mg excretion and blood Mg levels were not routinely monitored in these HIV patients [40]. However, there are a few clinical studies [20,22,41] which suggest a strong link between use of TDF-containing HAART and kidney dysfunction-mediated hypomagnesemia. Earlier studies [17,18,21] during the pre-HAART era have indicated that HIV-1 infection alone might also contribute to Mg-wasting. Findings from our current study suggest that both TDF administration and HIV-1 expression contributed synergistically to the observed hypomagnesemia.

Our published work [25,42,43,44] indicated that experimental hypomagnesemia alone can promote neurogenic (SP release) inflammation, partly through the Mg-gated *N*-methyl-d-aspartate (NMDA) receptor/calcium channel complex in neuronal tissue [25,45]. The neuropeptide SP is pro-inflammatory and pro-oxidative and plays a major role in causing oxidative stress [46] and cardiac contractile dysfunction [25,42,43]. We previously showed that the severity of diet-induced hypomagnesemia was directly proportional to the extent of circulating SP elevation and associated ROS stress in a rodent model [25]. In a subsequent study [42], echocardiography was used to demonstrate a link between chronic hypomagnesemia and left ventricular systolic and diastolic dysfunction in a rat model. In the current study, significantly elevated plasma SP levels were observed in the HIV-Tg rats receiving the cART treatment for 6–18 weeks. We also found that untreated Tg rats, which displayed modest hypomagnesemia (10–14% lower plasma levels versus control), had correspondingly modest increases in plasma SP levels throughout the 18-week experimental period. We speculate that the varying increases in circulating SP exhibited by both untreated Tg and more dramatically by cART-treated Tg rats may reflect, proportionately, the severity of systemic ROS/RNS stress, cardiac injury and dysfunction observed in these groups. In support of this notion, dietary Mg supplementation substantially suppressed the induced elevations of plasma SP in both untreated Tg and cART-treated Tg rats, and provided extensive improvement in cardiac hemodynamic and systolic and diastolic functional properties. Collectively, these findings of hypomagnesemia-induced SP elevations along with ROS/RNS-mediated cardiac injury and dysfunction in untreated Tg rats implicate a cardiotoxic synergism in Tg rats treated with cART.

## 4. Materials and Methods

### 4.1. HIV-1 Transgenic Rat Model, cART Treatment and Mg-Diets

Male 5 week-old Hsd:HIV-1 (F344) transgenic rats and the background wild type control (Fischer 344/NHsd) rats were obtained from Envigo/Harlan Laboratory (Indianapolis, IN, USA). Following 1 week quarantine, all rats were maintained under aseptic conditions in individual sterilized hepa-filtered isolator cages in a dedicated room by The George Washington University (GWU) Animal Research Facility (ARF). All rats were initially placed on an *ad libitum irradiated chow and sterilized water, and* were on a 12 h light/dark cycle for up to 7.5 months of age. All animal experiments were guided by the principles for the care and use of laboratory animals as recommended by the US Department of Health and Human Services and approved by The GWU Animal Care and Use Committee (9 June 2014; IACUC #A302). A description of the ARF can be obtained online at our GWU ARF website: http://research.gwu.edu/office-animal-research.

At 3 months old, the HIV-1 Tg and control rats were divided into 8 groups. Groups A1 to A4 were fed a normal Mg (0.1% Mg as MgO) diet, and groups B1 to B4 received a high Mg (0.6% Mg) diet [6,7,8,9] as follows: (A1) Control alone on normal Mg diet; (A2) Control + cART on normal Mg Diet; (A3) Tg alone on normal Mg diet; (A4) Tg + cART on normal Mg diet; (B1) Control alone on high Mg diet; (B2) Control + cART on high Mg diet; (B3) Tg on high Mg diet; and (B4) Tg + cART on high Mg diet. Both Mg diets, which contain extracted casein as the diet base and essential vitamins and nutrients, were irradiated and obtained from Envigo/Teklad, (Madison, WI, USA). The human fixed dosages of tenofovir (tenofovir disoproxil fumarate, TDF)/emtricitabine (FTC) in Truvada (Gilead Science Inc., Foster City, CA, USA) are 300 mg/200 mg/day which is equivalent to about 5.0/3.3 mg/kg/day; and for atazanavir (ATV, Bristol-Myers Squibb, New York, NY, USA)/ritonavir (RTV, Abbott Lab, Chicago, IL, USA) or ‘r’ if used as a lower boosting dose), the 300 mg/100 mg/day human dose is equivalent to about 5.0/1.65 mg/kg/day. Due to the higher drug metabolic rates in rodents [26], we designed the dosage to be about 3-fold higher than that of human on a per body weight basis. The levels of the drugs required to be mixed in the diet (performed by Teklad Lab, Madison, WI, USA) were estimated based on the known average daily food consumption data provided by Teklad; the quantities of the drug components added per kg diet were: 350 mg TDF, 230 mg FTC, 350 mg ATV, and 115 mg RTV. The exact daily dosages were calculated by the average daily amounts of diet consumed and were monitored on a weekly basis. The concurrent cART treatment while on normal Mg diet, or Mg supplemented diet was carried out for up to 18 weeks.

### 4.2. Blood Sample Collection

At 6-week intervals, blood (~0.5 mL was collected aseptically from the tail tip of anaesthetized rats (2% isoflurane, EZ Anesthesia Chamber plus nose cone) [47] in sterile microtainer plasma separator tubes containing sodium heparin and the protease inhibitor, aprotinin (Sigma Chemicals, St. Louis, MO, USA) yielding final blood concentrations of 10.74 units/mL and 0.016 units/mL, respectively. The scab was carefully removed, and the process was repeated for subsequent samplings. Plasma was obtained by centrifugation (12,000 rpm, 2 min, RT, IDEXX StatSpin VT, Iris International, Inc., Westbrook, ME, USA), and stored at −80 °C until used for assays. These samples were used for plasma Mg and substance P level assessments. Sacrifice blood (~8 mL collected in heparin plus aprotinin containing BD vacutainer SST tubes) was obtained from heparinized (0.3–0.4 mL 358 units/mL heparin in 0.9% NaCl, i.p.), euthanized rats via cardiac puncture, and samples were centrifuged (3500 rpm, 10 min, RT), and plasma samples were subsequently frozen (−80 °C) until assayed. In addition to the above plasma-derived parameters, sacrifice plasma was also assayed for 8-isoprostane and 3-nitrotyrosine levels, and the whole blood samples were processed for neutrophil isolation and assessment of superoxide anion production, as well as for RBC glutathione. At sacrifice, liver, kidney and heart tissue samples were rapidly excised, processed, frozen and stored at −80 °C.

### 4.3. Plasma Magnesium

Magnesium levels in 1:50 or 1:100 diluted, acidified (nitric acid) plasma samples were determined by flame emission atomic absorption spectroscopy (wavelength = 285.2 nm) using an AA-6200 Shimadzu spectrophotometer (Columbia, MD, USA) as described [7,8,9,47]. Values were obtained from standard curves as mg/dl, and reported as % of the control which was measured to be 19.6 ± 1.2 mg/dL at week 0.

### 4.4. Determination of Systemic Oxidative/Nitrosative Indices, Neutrophil ROS Activity, Plasma Creatinine, Urea and Substance P

Plasma 8-isoprostane levels were determined by enzyme immunoassay kits from Cayman Chemical (Ann Arbor, MI, USA) [7,8,9,43,47]. RBC reduced (GSH) and oxidized (GSSG) glutathione levels were assessed enzymatically by the DTNB-GSSG reductase method [7,8,9,47]. Plasma 3-nitrotyrosine levels were determined by the enzyme immunoassay kit from Cayman Chemical consisting of antiserum (anti-nitrotyrosine rabbit IgG) and Ellman’s Reagent. Neutrophils freshly isolated from whole blood were obtained from step-gradient centrifugation within 20 min of animal sacrifice [7,8,43,47]; both basal (no stimulus) and PMA-stimulated (125 ng/mL phorbol 12-myristate 13-acetate) superoxide anion production were estimated by SOD-inhibitable reduction of cytochrome c using the extinction coefficient: E550 = 2.1 × 10.4 M^−1^·cm^−1^. Plasma creatinine levels were determined by a kinetic colorimetric assay kit (Cayman Chemical). The assay is based on the Jaffe’s reaction method [48] in which a yellow-orange product is formed when the metabolite is reacted with alkaline picrate and was measured at 495 nm. The kinetic nature of the assay minimizes interference from contaminants such as bilirubin and lipids. Plasma urea levels were determined by the Urea Fluorometric Assay Kit (Cayman Chemical) [49], in which sample urea was hydrolyzed by urease into carbon dioxide and ammonia; the amount of ammonia generated reacts with the detector resulting in a fluorescent product analyzed with an excitation of 410 nm and an emission of 475 nm.

Plasma SP levels [25,42,47] were assessed using a competitive binding ELISA kit from R&D Systems (Minneapolis, MN, USA), in which SP, within samples (50 µL of 1:1 diluted plasma), competes with a fixed amount of horseradish peroxidase^−^ labeled SP for sites on a murine monoclonal antibody. Color development was inversely proportional to SP concentration and absorbance was read at 450 nm with background subtraction at 540 nm using a microplate reader (VersaMax, Molecular Devices, Sunnyvale, CA, USA). Values represent % changes in plasma SP levels compared to time-matched controls (100%) and were means ± SE of 4–6 rats. Overall average (*n* = 15) of control rat plasma SP levels at 6, 12 and 18 weeks was 541.64 ± 25 pg/mL and was within range of the R&D kit estimate.

### 4.5. Immunohistochemical Analysis of 3-Nitrotyrosine

Cardiac and kidney tissues were rapidly excised, rinsed in saline, embedded in OCT compound, frozen quickly and kept at −80 °C until used. Cryosections, 5 μm thick, were stained immunohistochemically [7,8,47] using rabbit anti-nitrotyrosine antibody (1:200; Millipore, Billerica, MA, USA) and the Vecta-Stain Elite ABC kit immunoperoxidase system (Vector Laboratories, Burlingame, CA, USA). Samples were examined under a Nikon Eclipse E600 microscope, and multiple images were taken with a digital camera (Spot Camera Diagnostic RT3 with advanced software Spot 5.3). Nitrotyrosine-positive areas were marked and percent of positive area within total tissue area in the microscopic field was obtained for 6 random fields along the kidney cortex and in cardiac ventricles using ImageJ software (Image processing and analysis in Java, http://imagej.nih.gov) in modified published procedures [47,50]. ImageJ is a reliable program for computer-assisted analysis in bright-field microscopy, provided that the micrographs can be easily converted into binary black and white images, which was readily achieved in our study. Briefly, 24-bit RGB images were converted into three 8-bit grayscale images by using the red-green-blue stack function and analyzed through the red channel, where contrast between the area of nitrotyrosine location and the background was the greatest. The number of pixels in the entire tissue area was obtained first. Then, by selecting the “Image-Adjust-Threshold” function, the threshold was set and manually adjusted to mark in red the areas of interest. When the satisfying threshold levels were set, the number of pixels in the area included within the threshold was determined through ImageJ’s “Measure” function, and reported as a percent positive for nitrotyrosine area versus total tissue area in the microscopic field. The brown staining in hearts is predominantly localized in perivascular connective tissue and occasionally in smooth muscle cells of intima tunica and in endothelial cells. Brown staining for nitrotyrosine in kidney appeared more diffused in the entire cortex area; therefore, for publication purposes, it was more advantages to present the results using the images obtained from ImageJ software analysis, in which points of interest are marked in red color instead in brown.

### 4.6. Non-Invasive Ultra-High Frequency Echocardiography

Three-month-old rats were subjected to the treatment regimens and echocardiography was performed [6,8,42,43,47] at 0 (baseline), 6, 12, and 18 weeks of treatment. This procedure was performed in an aseptic positive-pressure laminar flow hood within the dedicated ARF holding room. Echocardiography (GE VingMed System Five Echocardiogram System (GE Medical System, Hanover, MD, USA) was performed on the anesthetized rats using 2% inhaled isoflurane mixed with 95% O_2_/5% CO_2_ (EZ™ anesthesia chamber with nose cone). Heart rate and rectal temperature (35.9–37.5 °C) were monitored during imaging. Animals were placed on a warming platform with paws taped down to the platform to improve access to the thoracic area for electrocardiogram monitoring. A sterile eye lubricant was applied to prevent drying during the procedure. After shaving with an electric razor, remaining hair over the thorax was removed with a depilatory cream (<2 min exposure). Animals were imaged (10 MHz probe) for 20 min at a 3 cm image depth. The resolution of this system allows for both cardiac structure and function evaluation in rats. Left ventricular systolic function, measured as % fractional shortening (%FS) using M-mode imaging, and left ventricular ejection fraction (LVEF), calculated from M-mode measurements, were assessed. Key anatomical parameters (IVSd & s = inter-ventricular septum diameter in diastole and systole; LVDd & LVDs = LV internal chamber diameter in diastole and systole; and LVPWd & s = LV posterior wall thickness in diastole or systole) were measured, and the presence of dilated or hypertrophic cardiomyopathy assessed. Aortic and pulmonary artery diameter measurements were used to calculate stroke volumes. Aortic pressure maximum and mean (AoPmax & mean) were determined, and spectral Doppler velocities were measured for the pulmonic and aortic outflows to calculate cardiac output (CO), and for the mitral valve inflows to assess ventricular diastolic function (mitral valve E and A wave velocities and the E/A ratio). Inflow pattern changes are seen with diastolic dysfunction. Prior to initiating the treatment regimens (baseline), echocardiography did not reveal significant differences between HIV-Tg and control rats at age 3 months (Figure S1, Tables S1 and S2). Following echocardiography, rats were placed in ambient air, observed for 1 h until fully recovered and then returned to ARF holding cages.

### 4.7. Statistics

Data, i.e., means ± SEM of 5 rats per group, were assessed by *F*-test for equality of group variation and then by two-tailed Student’s *t*-test to evaluate statistical differences. One-way ANOVA followed by a Tukey’s test were used to analyze selected data. Values of *p* < 0.05 were considered statistically significant.

## 5. Conclusions

**Figure 11:**
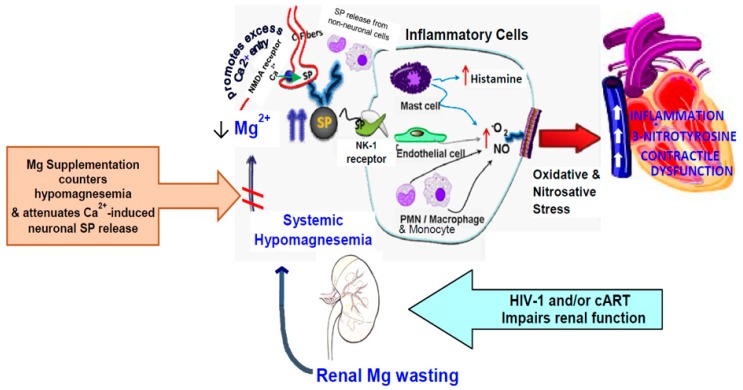
Chronic use of cART in HIV-1 infected patients may impair renal function, leading to magnesuria (Mg wasting) and hypomagnesemia-induced neurogenic (substance P [SP]) inflammation. Increased circulating SP levels, initially via activation of Mg-gated neuronal NMDA receptor (C-fibers), result in elevated inflammatory mediators (cytokines), ROS/RNS production, antioxidant depletion, and further kidney, and cardiac pathology/dysfunction. We suggest that chronic oxidative/nitrosative stress associated with prolonged hypomagnesemia may amplify and/or sustain SP-mediated injury. Mg supplementation may lessen the hypomagnesemic-inducing effects caused by cART in HIV patients, and should reduce the risk of kidney, and cardiac toxicity/dysfunction by limiting SP bioavailability.

In conclusion, by using an HIV-1 transgenic rat model, we demonstrated that a relatively low human equivalent dose of cART regimen can cause significant systemic and cardiac oxidative/nitrosative stress, leading to moderate, yet significant cardiac dysfunction. To our knowledge, this is the first report describing a causative impact of HIV-1 expression with or without cART treatment on moderate but significant degrees of hypomagnesemia and cardiac dysfunction in this model. Our evidence further suggests that the severity of hypomagnesemia and the corresponding elevations in circulating SP likely occurred secondary to the extent of renal Mg wasting. The proposed schematic steps of cART-induced neurogenic/oxidative inflammation under HIV-1 infection resulting in enhanced cardiac dysfunction, and the Mg protective mechanisms are summarized by Figure 11 (Graphic representation). In light of reports that blood SP levels were elevated in HIV-infected men [51] and women [52], and that a large subpopulation of HIV-1 infected individuals (~30%) also have co-existing hypomagnesemia [17,18,19,20,21], we state that our findings with HIV-1 transgenic rats indicate the significant clinical relevance of this model. Of equal importance, Mg-supplementation completely prevented the development of kidney dysfunction, lessened the elevations of circulating SP, and substantially protected against the cardiac dysfunction in both untreated and cART-treated Tg rats; these findings suggest a potential adjunct therapeutic role for Mg supplements during cART treatment of HIV patients. Since TDF containing Truvada remains the preferred backbone NRTIs for current cART regimens and has also been recommended prophylactically since 2012 to prevent HIV-1 pre-exposure, it is likely that Truvada will continue to be used globally for years to come. From this perspective, our study suggest that Mg-supplementation can be used as an effective, yet economical co-therapy to attenuate adverse cardio-renal side effects caused by Truvada-containing cART treatment of HIV-infected individuals.

## Figures and Tables

**Table 1:** Effect of cART treatment for 18 weeks on food consumption and weight gain of control and HIV-Tg rats receiving normal Mg or Mg supplemented diets.

	Initial Wt	Wt Gain at 18 Weeks	Food Consumed	cART Dosage
Rat Groups	(gm)	(% of Initial wt)	(gm/kg/day)	(mg/kg/day)
**Normal Mg**				
A-1 (Ctl)	269 ± 8	38.1 ± 3.1	41.7 ± 2.1	
A-2 (+cART)	267 ± 6	38.4 ± 3.7	43.1 ± 2.1	47.5 ± 2.3
A-3 (Tg)	236 ± 4	38.9 ± 1.3	44.0 ± 1.8	
A-4 (Tg + cART)	261 ± 5	29.4 ± 0.8 **	42.7 ± 1.7	47.3 ± 1.9
**Mg Supplemented**				
B-1 (Ctl)	260 ± 8	39.2 ± 3.4	44.6 ± 6.0	
B-2 (+cART)	249 ± 7	45.1 ± 2.2	43.3 ± 2.7	47.7 ± 2.7
B-3 (Tg)	229 ± 9	49.0 ± 4.2 *	46.6 ± 6.5	
B-4 (Tg + cART)	219 ± 6	48.2 ± 2.0 **^++^**	43.8 ± 3.7	48.1 ± 3.8

Data were based on an average of 5 ± SEM; * *p* < 0.05, ** *p* < 0.01 vs. Controls, ^++^ *p* < 0.01 vs. A-4. Mg diet and drug dose information can be found in Section 4.

## Supplementary Materials

Supplementary materials can be found at http://www.mdpi.com/1422-0067/19/8/2409/s1.
