# Supplementary material for: Combination ART-Induced Oxidative/Nitrosative Stress, Neurogenic Inflammation and Cardiac Dysfunction in HIV-1 Transgenic (Tg) Rats: Protection by Mg
Source: Int J Mol Sci. 2018 Aug 15;19(8):2409. doi: 10.3390/ijms19082409 (PMC6121319; doi:10.3390/ijms19082409)
Supplement: Supplementary file 1 [file ijms-19-02409-s001.zip › ijms-328649-supplementary.pptx]

## Slide 1
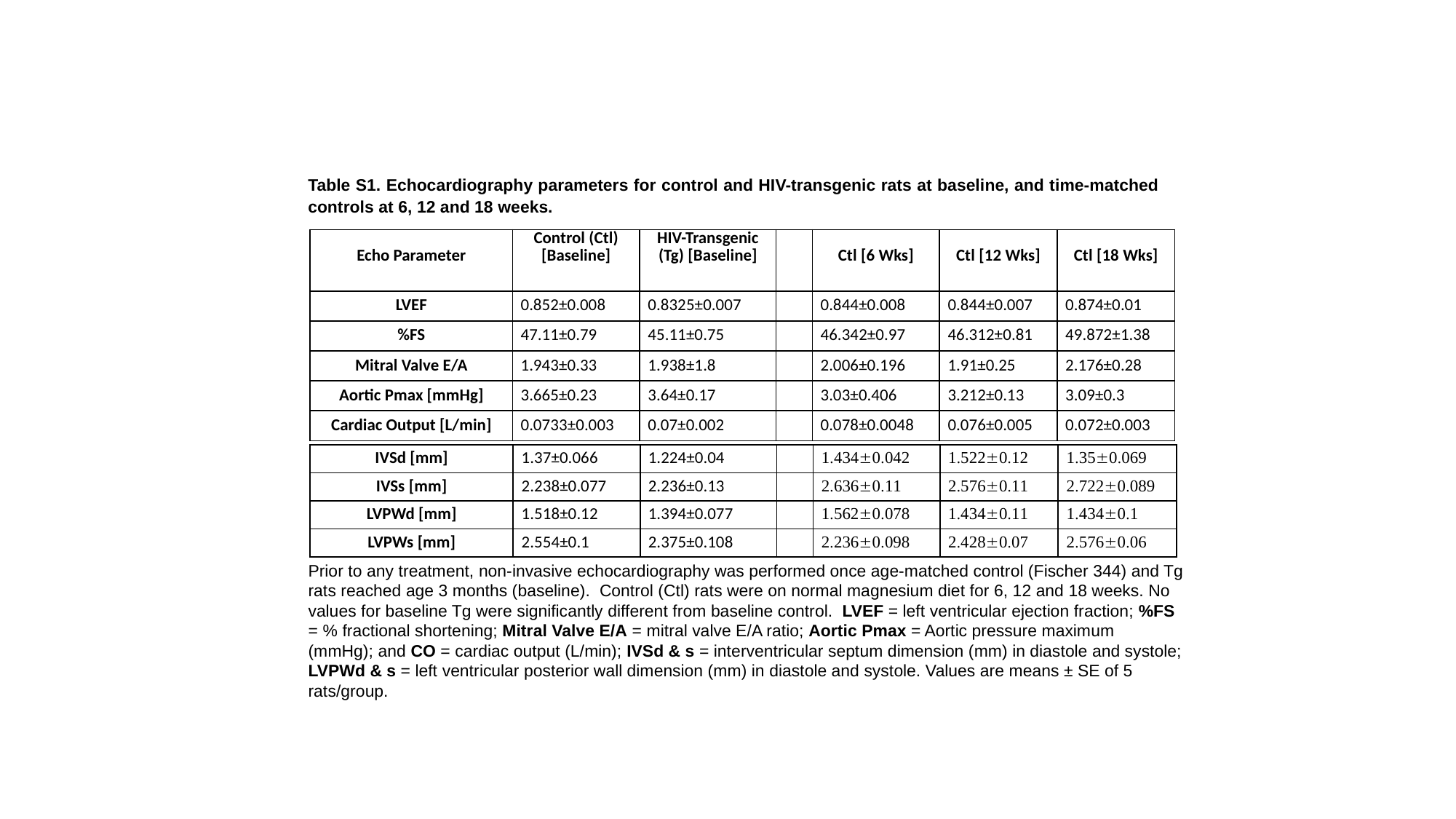

Table S1. Echocardiography parameters for control and HIV-transgenic rats at baseline, and time-matched controls at 6, 12 and 18 weeks.
| Echo Parameter | Control (Ctl) [Baseline] | HIV-Transgenic (Tg) [Baseline] | | Ctl [6 Wks] | Ctl [12 Wks] | Ctl [18 Wks] |
| --- | --- | --- | --- | --- | --- | --- |
| LVEF | 0.852±0.008 | 0.8325±0.007 | | 0.844±0.008 | 0.844±0.007 | 0.874±0.01 |
| %FS | 47.11±0.79 | 45.11±0.75 | | 46.342±0.97 | 46.312±0.81 | 49.872±1.38 |
| Mitral Valve E/A | 1.943±0.33 | 1.938±1.8 | | 2.006±0.196 | 1.91±0.25 | 2.176±0.28 |
| Aortic Pmax [mmHg] | 3.665±0.23 | 3.64±0.17 | | 3.03±0.406 | 3.212±0.13 | 3.09±0.3 |
| Cardiac Output [L/min] | 0.0733±0.003 | 0.07±0.002 | | 0.078±0.0048 | 0.076±0.005 | 0.072±0.003 |
| IVSd [mm] | 1.37±0.066 | 1.224±0.04 | | 1.434±0.042 | 1.522±0.12 | 1.35±0.069 |
| --- | --- | --- | --- | --- | --- | --- |
| IVSs [mm] | 2.238±0.077 | 2.236±0.13 | | 2.636±0.11 | 2.576±0.11 | 2.722±0.089 |
| LVPWd [mm] | 1.518±0.12 | 1.394±0.077 | | 1.562±0.078 | 1.434±0.11 | 1.434±0.1 |
| LVPWs [mm] | 2.554±0.1 | 2.375±0.108 | | 2.236±0.098 | 2.428±0.07 | 2.576±0.06 |
Prior to any treatment, non-invasive echocardiography was performed once age-matched control (Fischer 344) and Tg rats reached age 3 months (baseline). Control (Ctl) rats were on normal magnesium diet for 6, 12 and 18 weeks. No values for baseline Tg were significantly different from baseline control. LVEF = left ventricular ejection fraction; %FS = % fractional shortening; Mitral Valve E/A = mitral valve E/A ratio; Aortic Pmax = Aortic pressure maximum (mmHg); and CO = cardiac output (L/min); IVSd & s = interventricular septum dimension (mm) in diastole and systole; LVPWd & s = left ventricular posterior wall dimension (mm) in diastole and systole. Values are means ± SE of 5 rats/group.

## Slide 2
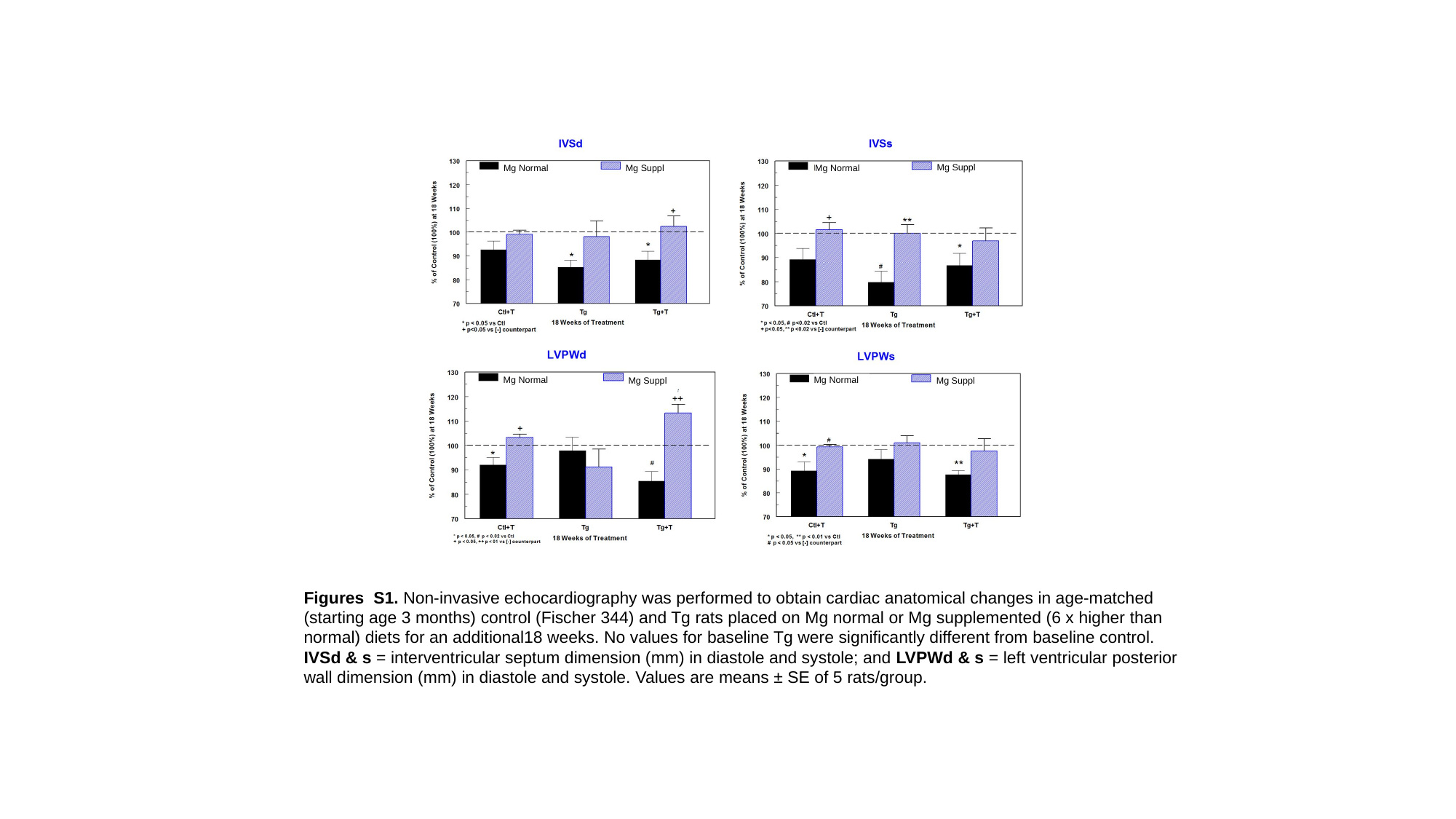

Mg Suppl
Mg Normal
Mg Normal
Mg Suppl
Mg Normal
Mg Suppl
Mg Normal
Mg Suppl
Figures S1. Non-invasive echocardiography was performed to obtain cardiac anatomical changes in age-matched (starting age 3 months) control (Fischer 344) and Tg rats placed on Mg normal or Mg supplemented (6 x higher than normal) diets for an additional18 weeks. No values for baseline Tg were significantly different from baseline control. IVSd & s = interventricular septum dimension (mm) in diastole and systole; and LVPWd & s = left ventricular posterior wall dimension (mm) in diastole and systole. Values are means ± SE of 5 rats/group.

## Slide 3
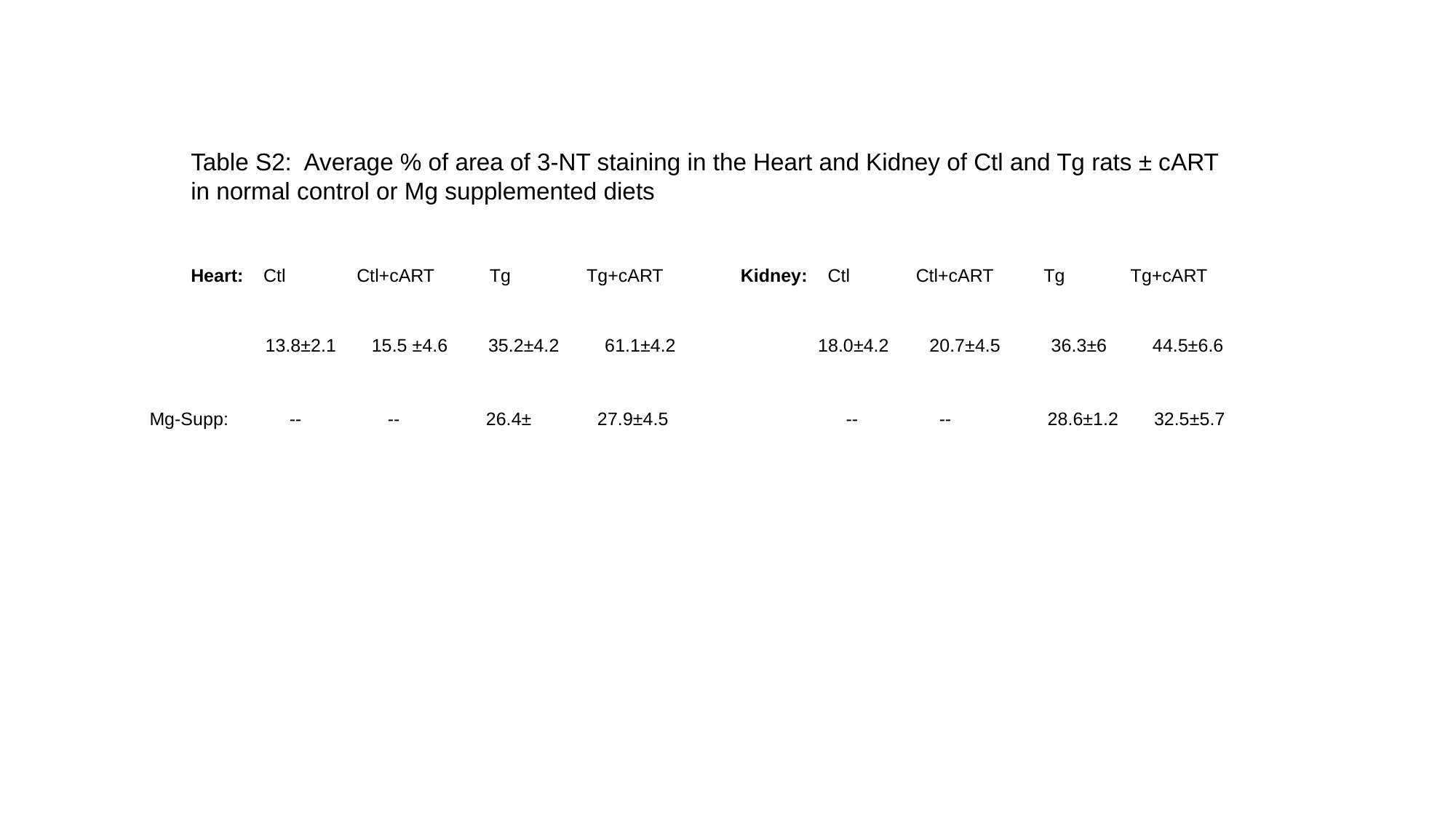

Table S2: Average % of area of 3-NT staining in the Heart and Kidney of Ctl and Tg rats ± cART in normal control or Mg supplemented diets
Heart: Ctl Ctl+cART Tg Tg+cART Kidney: Ctl Ctl+cART Tg Tg+cART
 13.8±2.1 15.5 ±4.6 35.2±4.2 61.1±4.2 18.0±4.2 20.7±4.5 36.3±6 44.5±6.6
Mg-Supp: -- -- 26.4± 27.9±4.5 -- -- 28.6±1.2 32.5±5.7
